# Investigating Nonlinear Fatigue Damage Evolution of SBS-Modified Asphalt Mixtures with Physical Gel Structure

**DOI:** 10.3390/gels12060559

**Published:** 2026-06-22

**Authors:** Chenze Fang, Yuanzhao Chen, Yi Lu, Zhenxia Li, Hui Li, Xu Guo, Jingyu Yang, Tengteng Guo

**Affiliations:** 1 School of Civil Engineering and Transportation, North China University of Water Resources and Electric Power, Zhengzhou 450045, China; 2 Technology Innovation Center of Henan Transport Industry of Utilization of Solid Waste Resources in Trafffc Engineering, North China University of Water Resources and Electric Power, Zhengzhou 450045, China; 3 Henan Province Engineering Technology Research Center of Environment Friendly and High-Performance Pavement Materials, Zhengzhou 450045, China; 4 School of Foreign Languages, North China University of Water Resources and Electric Power, Zhengzhou 450045, China; 5 Department of Transportation, Southeast University, Nanjing 210096, China

**Keywords:** pavement engineering, SBS-modified asphalt mixture, residual strain, damage model, fatigue damage evolution

## Abstract

Styrene-butadiene-styrene (SBS) modifier can enhance the resistance of asphalt mixtures to load-induced deformation and fatigue cracking by constructing a three-dimensional physical gel network. However, a rigorous mechanical characterization of this mechanism remains lacking. This study elucidates the nonlinear fatigue damage evolution of SBS-modified asphalt mixtures with physical gel structures based on residual strain response analysis. Indirect tensile fatigue tests were conducted to characterize the residual strain response of SBS-modified asphalt mixtures. A damage-informed residual strain model was established, and a relative residual strain change rate was defined to analyze the correlation between fatigue cracking and residual strain response. Furthermore, the nonlinear fatigue damage evolution of SBS-modified asphalt mixtures was investigated based on the fatigue damage theory. The results demonstrate a strong correlation between fatigue cracking and a viscoplastic strain in the SBS-modified asphalt mixtures. The proposed residual strain model accurately describes the nonlinear fatigue damage evolution and residual strain response. The relative residual strain change rate serves as a rational indicator of the material’s resistance to fatigue cracking and residual strain accumulation. The SBS modifier enhances resistance to residual strain and fatigue cracking by forming a complex polymer network that establishes a three-dimensional physical gel structure.

## 1. Introduction

Styrene-butadiene-styrene (SBS)-modified asphalt mixtures have been widely adopted in pavement engineering owing to their superior resistance to high-temperature rutting, low-temperature cracking, and fatigue damage [1,2,3]. The SBS modifier forms a three-dimensional polymer network within the asphalt matrix through physical crosslinking, establishing a physical gel architecture that endows the material with remarkable elastic recovery and deformation compatibility, thereby substantially enhancing its mechanical performance and durability [4,5]. Nevertheless, under sustained cyclic vehicular loading, SBS-modified asphalt mixtures inevitably accumulate internal fatigue damage, which progressively culminates in fatigue cracking and compromises pavement service life [6,7]. Among various polymer modifiers, styrene-butadiene-styrene (SBS) triblock copolymer is selected in this study because it is the most commonly used modifier in pavement engineering. SBS-modified asphalt has been widely used in paving asphalt pavement all over the world and a thermally reversible physical gel network is formed by microphase separation of polystyrene blocks [8]. Compared with diblock or statistical copolymers with the same chemical composition, the triblock structure can achieve more effective physical crosslinking, thereby obtaining better mechanical properties [9]. Consequently, a comprehensive understanding of the fatigue damage evolution mechanisms in SBS-modified asphalt mixtures is of critical importance for optimizing material design and extending pavement longevity.

Existing studies have established that the fatigue performance of SBS-modified asphalt mixtures is intrinsically governed by their viscoelastic response characteristics [10,11]. Under cyclic loading conditions, fatigue damage and residual strain accumulation concurrently evolve within the material, exhibiting a pronounced phenomenological correlation [12,13,14]. Employing digital image correlation techniques, Jiang et al. [15,16] demonstrated that residual strain and fatigue crack propagation in asphalt mixtures follow analogous three-stage evolution patterns, enabling the tracking of fatigue damage progression through residual strain analysis. Motivated by these findings, residual strain-based methodologies have been developed to characterize fatigue damage. Zhang et al. [17,18] formulated a mechanical model based on a modified Burgers framework, decomposing residual strain into viscoelastic and viscoplastic components, thereby providing a theoretical foundation for quantitative damage assessment. Furthermore, Fang et al. [19,20] introduced a relative rate of residual strain variation, which exhibits strong correlation with fatigue life and effectively captures the material’s resistance to fatigue cracking.

Despite extensive research on residual strain response and fatigue damage evolution in base asphalt mixtures and crumb rubber-modified asphalt mixtures, systematic investigations specifically targeting styrene-butadiene-styrene (SBS)-modified asphalt mixtures remain notably scarce. Compared with base asphalt, SBS-modified asphalt exhibits substantially more complex viscoelastic mechanical behavior, primarily attributable to the three-dimensional physical gel network formed by the SBS modifier within the asphalt matrix. On one hand, this physically crosslinked network enhances the material’s elastic recovery capacity, enabling greater recovery of residual viscoelastic strain upon unloading [21,22]. On the other hand, the physical gel structure significantly improves the material’s resistance to viscoplastic deformation, thereby delaying the accumulation of residual strain [23,24]. These distinct mechanical characteristics dictate that the fatigue damage evolution law of SBS-modified asphalt mixtures fundamentally differs from that of base asphalt mixtures. Furthermore, existing residual strain models largely neglect the nonlinear influence of fatigue damage on the material’s mechanical behavior, rendering them incapable of accurately characterizing the accelerated failure features during damage progression [25,26]. It should be emphasized that the three-dimensional physical gel network and entanglement structure of SBS-modified asphalt have been fully verified in previous literature, which is a mature consensus in pavement engineering. This paper focuses on macroscopic mechanical response and fatigue damage modeling rather than microstructural characterization [1,2,3,27].

Therefore, this study aims to systematically elucidate the nonlinear fatigue damage evolution of SBS-modified asphalt mixtures based on residual strain response analysis. First, indirect tensile fatigue tests are conducted under different temperature conditions to characterize the residual strain response features of SBS-modified asphalt mixtures. Second, within the framework of viscoelastic fatigue damage theory, a residual strain model incorporating damage effects is established to achieve a synergistic characterization between residual strain response and fatigue damage evolution. Third, the correlation between fatigue cracking and residual strain response is analyzed by defining a relative residual strain rate. Finally, based on continuum fatigue damage mechanics, the nonlinear fatigue damage evolution law of SBS-modified asphalt mixtures is revealed, and the underlying mechanism by which the three-dimensional physical gel network formed by the SBS modifier enhances the fatigue performance of the material is clarified. The findings of this study are expected to provide a theoretical basis for the fatigue performance evaluation and design of SBS-modified asphalt pavements.

## 2. Results and Discussion

### 2.1. Establishment of Residual Strain Model for SBS-Modified Asphalt Mixture Considering Damage

#### 2.1.1. Analysis of Residual Strain Results

**Figure 1:**
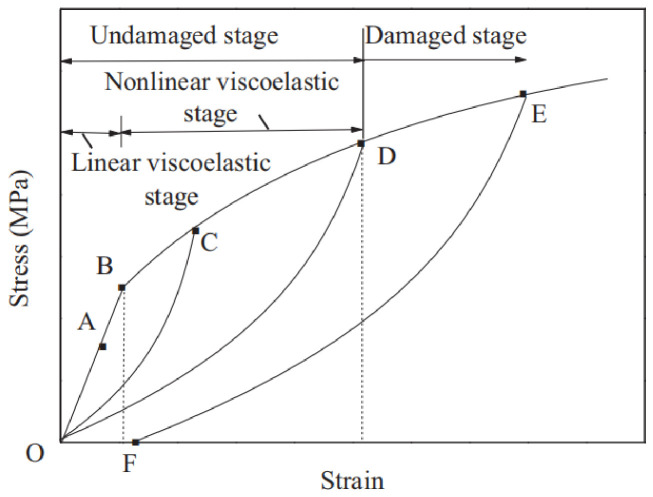
Different material stages of asphalt mixtures.

Theoretically, the material stage of asphalt mixture consists of an undamaged stage and a damage stage. The undamaged stage includes the linear viscoelastic stage and the nonlinear viscoelastic stage, as shown in Figure 1. Asphalt mixtures exhibit different material characteristics at different material stages [28]:

(1) When the load level is very low, the asphalt mixture is in the linear viscoelastic stage (OB). In this stage, the loading path (OA) and unloading path (AO) coincide, and the deformation can be fully recovered after unloading. No residual strain or damage occurs.

(2) As the load increases further, the asphalt mixture enters the nonlinear viscoelastic stage (BD). Although the loading path (OBC) and unloading path (CO) do not coincide in this stage, the deformation can still be fully recovered, and no damage occurs.

(3) As the load continues to increase, the asphalt mixture enters the damage stage (DE). In this stage, the loading path (OBE) and unloading path (EF) do not coincide. After unloading, the deformation cannot be fully recovered, leading to the accumulation of residual strain and the occurrence of damage.

**Figure 2:**
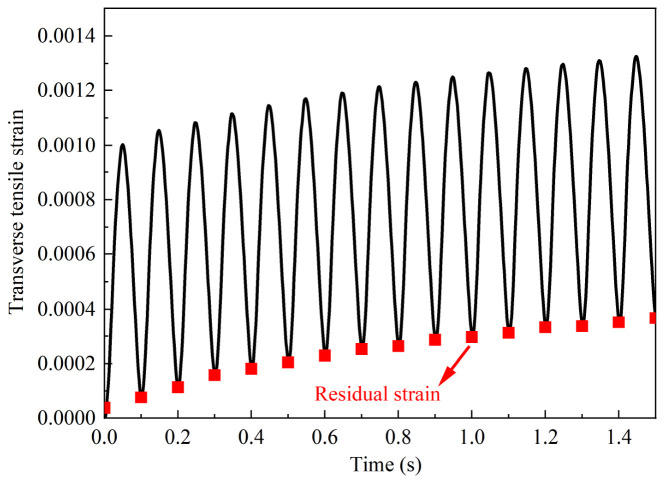
Strain curves of indirect tensile fatigue test.

Under cyclic loading, the total strain of asphalt mixture is mainly composed of elastic strain, viscoplastic strain, and viscoelastic strain. After unloading, the elastic strain is fully recovered, the viscoplastic strain cannot be recovered, and part of the viscoelastic strain is recoverable. Figure 2 shows the measured transverse tensile strain of the SBS-modified asphalt mixture, where the strain at the valley of each cycle is taken as the residual strain. As shown in Figure 2, the transverse tensile strain curve of the asphalt mixture under cyclic loading exhibits an overall rising cyclic waveform. This is because the applied cyclic load places the asphalt mixture in the damage stage, causing the loading and unloading paths to not coincide. The viscoplastic strain accumulates, and part of the viscoelastic strain cannot be fully recovered at the end of each cycle, leading to a continuous accumulation of residual strain at the valley of each cycle.

**Figure 3:**
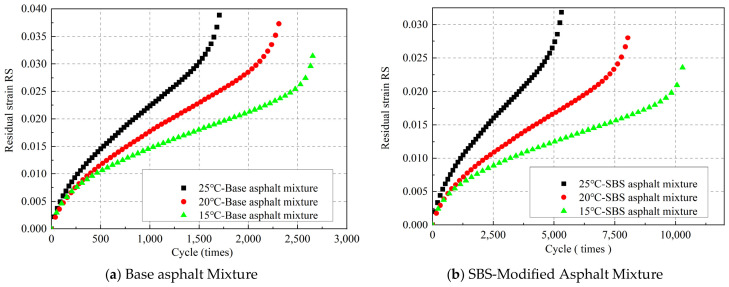
Residual strain of asphalt mixtures under different temperatures.

The residual strain results of SBS-modified and base asphalt mixtures at different temperatures are shown in Figure 3. As can be seen from Figure 3, the nonlinear residual strain evolution of asphalt mixtures consists of three stages. When the temperature gradually increases from 15 °C, the residual strain rate shows a significant upward trend. This is because at lower temperatures, the asphalt mixture can withstand more load cycles without viscous flow, resulting in a smaller residual strain rate. As the temperature increases, the viscoplastic deformation generated by each load cycle increases, causing the residual strain rate to be positively correlated with temperature. In addition, the residual strain rate of SBS-modified asphalt mixture is lower than that of the base asphalt mixture. This is because the SBS modifier forms a three-dimensional physical gel structure within the overall material structure of the asphalt mixture, enhancing its deformation resistance.

#### 2.1.2. Establishment of Fatigue Damage Model

Research by Academician Zheng Jianlong’s team [29,30] has shown that using a sine function σ(t)=σsinωt can reasonably simulate cyclic vehicle loads on pavements. By analyzing the evolution of material energy dissipation, the damage evolution behavior of asphalt and asphalt mixtures can be described with a relatively high accuracy. The strain response generated by the sine load is:

(1) $$\varepsilon (t)=\left|J*\right|\sigma \sin(\omega t+\varphi )=\left|J*\right|\sigma (\cos\omega t\sin\varphi +\sin\omega t\cos\varphi )$$

where σ is the stress amplitude; $\left|J*\right|$ is the creep compliance; ω is the angular frequency; and φ is the phase angle.

The dissipated energy (Δ*W_n_*) generated in the nth cycle can be expressed as:

(2) $$\begin{array}{ll} \Delta W_{n} & =\int_{T_{n-1}}^{T_{n}}\varepsilon (t)d\sigma (t)=\int_{T_{n-1}}^{T_{n}}\left|J*\right|\sigma^{2}\omega (\sin\omega t\cos\omega t\cos\varphi +\cos^{2}\omega t\sin\varphi )dt\\ \; & =\int_{T_{n-1}}^{T_{n}}\left[\left|J_{n-1}^{*}\right|+\frac{\left|J_{n}^{*}\right|-\left|J_{n-1}^{*}\right|}{T_{n}-T_{n-1}}(t-T_{n-1})\right]\sigma^{2}\omega (\sin\omega t\cos\omega t\cos\varphi +\cos^{2}\omega t\sin\varphi )dt\\ \; & =\frac{\sigma^{2}}{4}(J_{1_{n-1}}-J_{1_{n}})+\pi \sigma^{2}{\bar{J}}_{2_{n}}\\ \; & =W_{n1}+W_{n2} \end{array}$$

in which $J_{1_{n}}$ denotes the result of the storage compliance at the end of the *T_n_*_−1_ cycle; $J_{1_{n-1}}$ represents the result of the storage compliance at the end of the *T_n_* cycle; ${\bar{J}}_{2_{n}}$ corresponds to the average value of the dissipation compliance over the cycle interval *T_n_*_−1_ to *T_n_*; *W_n_*_1_ represents the elastic energy dissipation within the Nth cycle, while *W_n_*_2_ refers to the viscous energy dissipation within the same cycle.

The cumulative dissipated energy corresponding to the end of the Nth cycle is:

(3) $$\begin{array}{cc} W_{N} & =\sum_{n=1}^{N}W_{n}=\sum_{n=1}^{N}W_{n1}+\sum_{n=1}^{N}W_{n2}=W_{1N}+W_{2N}\\ \; & =\frac{\sigma^{2}}{4}(J_{1_{0}}-J_{1_{N}})+\sum_{n=1}^{N}\sigma^{2}M \end{array}$$

where $W_{N}$ is the cumulative dissipated energy at the end of the Nth cycle; $W_{n}$ is the dissipated energy generated within the nth cycle; $W_{1N}$ is the cumulative elastic energy dissipation at the end of the Nth cycle; $W_{2N}$ is the cumulative viscous energy dissipation at the end of the Nth cycle; $M=\pi {\bar{J}}_{2_{n}}$.

Since the fatigue failure of the asphalt mixture specimen occurs, *W*_1*N*_ is much smaller than *W*_2*N*_, thus, the cumulative dissipated energy corresponding to the end of the Nth cycle can be reasonably simplified as:

(4) $$W_{N}=\sum_{n=1}^{N}\sigma^{2}M$$

Based on strain equivalence, fatigue damage can be introduced into the above equation, yielding:

(5) $$W_{N}=\sum_{n=1}^{N}\frac{\sigma^{2}M}{{\left(1-D\right)}^{2}}$$

It can be seen from the above equation that:

(6) $$\frac{\partial W_{N}}{\partial N}=\frac{\sigma^{2}M}{{\left(1-D\right)}^{2}}$$

The damage evolution equation characterized by dissipated energy can describe the fatigue damage evolution behavior of viscoelastic materials with relatively high accuracy, as follows:

(7) $$\frac{dD}{dN}={\left(\frac{\partial W_{N}}{\partial N}\right)}^{\beta }$$

where *β* is a viscoelastic material parameter dependent on factors such as temperature, stress amplitude, frequency, and aging.

From the above equation, it can be obtained that:

(8) $$\frac{dD}{dN}=(\sigma^{2}M{)}^{\beta }{\left(1-D\right)}^{-2\beta }$$

By integrating the above equation over the interval *D* ∈ [0, 1) and *N* ∈ [0, *N_f_*), *N* can be obtained as:

(9) $$N=\frac{{\left(\sigma^{2}M\right)}^{-\beta }}{1+2\beta }-\frac{{\left(\sigma^{2}M\right)}^{-\beta }{\left(1-D\right)}^{1+2\beta }}{1+2\beta }$$

By integrating the above equation over the interval *D* ∈ [0, 1] and *N* ∈ [0, *N_f_*], *N_f_* can be obtained as:

(10) $$N_{f}=\frac{{\left(\sigma^{2}M\right)}^{-\beta }}{1+2\beta }$$

By taking the ratio of *N* to *N_f_*, the viscoelastic fatigue damage model can be obtained as:

(11) $$D=1-(1-\frac{N}{N_{f}}{)}^{\frac{1}{1+2\beta }}$$

The structure of this model is straightforward, involving a single parameter *β*, which enhances both theoretical derivation and practical applicability. Consequently, this study will employ the model to investigate the fatigue damage characteristics of asphalt mixtures.

#### 2.1.3. Establishment of Residual Strain Model Considering Damage

The evolution of fatigue damage accelerates the accumulation of residual strain in asphalt mixtures, and the continued accumulation of residual strain, in turn, influences the evolution of fatigue damage. When establishing the residual strain model, the impact of fatigue damage must be considered. Residual strain is primarily composed of viscoplastic strain and the irrecoverable portion of viscoelastic strain.

**Figure 4:**
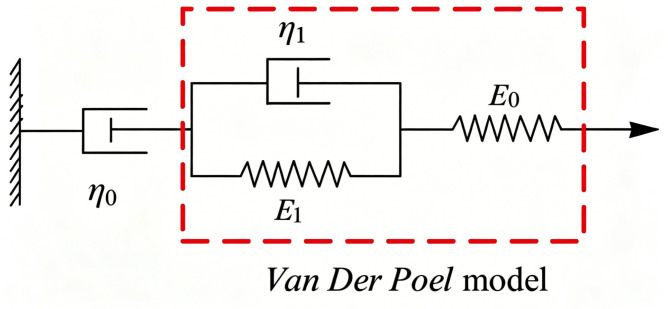
Structural analysis of Burgers model.

The Burgers model can accurately characterize the different types of strain in asphalt mixtures and has been widely applied in road engineering. Its mechanical model derivation process is relatively simple and conducive to generalization. Therefore, this study intends to adopt the Burgers model to establish a residual strain mechanical model suitable for asphalt mixtures. As shown in Figure 4, the Burgers model consists of a series of combinations of a dashpot (*η*_0_) and a three-element VanDerPoel model.

In the conventional Burgers model, the viscosity coefficient of the series dashpot is constant. To better capture the nonlinear mechanical response of asphalt mixtures, researchers have proposed the following exponential modification of the series dashpot:

(12) $$\eta_{0}(t)=Ae^{Bt}$$

where *η*_0_(*t*) represents the viscosity coefficient of the series dashpot at time (t), and (A) and (B) are model parameters. Although this model can accurately describe the residual strain behavior in the first two stages, it fails to accurately capture the residual strain behavior in the third stage.

Relevant studies [31] indicate that by applying a bivariate function modification to the series dashpot, as shown in the following equation, the variation in residual strain across all three stages can be described more comprehensively and accurately.

(13) $$\eta_{0}(t)=\frac{\eta_{0}}{at^{2}+bt+1}$$

in which a and b are constants, where a > 0, b < 0, and b2−4a < 0; η_0_ is the initial viscosity coefficient.

In this study, the fatigue tests on asphalt mixtures were conducted under a half-sine cyclic load with an amplitude of *σ*_0_ and a period of *T*. The viscosity coefficient of the series dashpot in the (i)-th cycle (*η*_0,*i*_) is given by:

(14) $$\eta_{0,i}=\frac{\eta_{0}}{a{\left[(i-1)T+\tau \right]}^{2}+b[(i-1)T+\tau ]+1}$$

where *τ* represents the load application time in the i-th cycle.

According to viscoelastic theory, the accumulated viscoplastic strain in the (i)-th cycle (ɛ_vp_,*_i_*) is given by:

(15) $$\begin{array}{cc} \varepsilon_{\mathrm{vp},i}= & \int_{0}^{T}\frac{\sigma (\tau )}{\eta_{0,i}(\tau )}d\tau =\frac{2a\sigma_{0}T^{3}}{\pi \eta_{0}}{\left(i-1\right)}^{2}+\;\frac{2b\sigma_{0}T^{2}+2a\sigma_{0}T^{3}}{\pi \eta_{0}}(i-1)\\ \; & +\;\frac{\sigma_{0}}{\pi \eta_{0}}(2T+bT^{2}+aT^{3}-\frac{4aT^{3}}{\pi^{2}}) \end{array}$$

After (N) cycles, the accumulated viscoplastic strain (ɛ_vp_, N) is given by:

(16) $$\begin{array}{cc} \varepsilon_{\mathrm{vp},N}= & \sum_{i=1}^{N}\varepsilon_{\mathrm{vp},i}\;=\frac{2a\sigma_{0}T^{3}}{\pi \eta_{0}}\frac{N(N-1)(2N-1)}{6}+\frac{2b\sigma_{0}T^{2}+2a\sigma_{0}T^{3}}{\pi \eta_{0}}\frac{N(N-1)}{2}\\ \; & +\frac{\sigma_{0}}{\pi \eta_{0}}(2T+bT^{2}+aT^{3}-\frac{4aT^{3}}{\pi^{2}})N \end{array}$$

The creep compliance of the Van der Poel model at time (t) is given by:

(17) $$J(t)=\frac{1}{E_{0}}+\frac{1}{E_{1}}(1-e^{-E_{1}t/\eta_{1}})$$

where *E*_0_ is the elastic modulus of the series spring; *E*_1_ is the elastic modulus of the parallel spring; and *η*_1_ is the viscosity coefficient of the parallel dashpot. According to the Boltzmann superposition principle, the accumulated residual viscoelastic strain in the i-th cycle (*ɛ*_rve,_ *_i_*) is given by:

(18) $$\varepsilon_{\mathrm{rve},i}=\int_{0}^{T}J\left[NT-(i-1)T-\tau \right]\frac{d\sigma (\tau )}{d\tau }d\tau =\frac{\pi \sigma_{0}T(1+e^{E_{1}T/\eta_{1}})}{\eta_{1}(\frac{E_{1}^{2}}{\eta_{1}^{2}}T^{2}+\pi^{2})}e^{-\frac{E_{1}}{\eta_{1}}\left[N-(i-1)\right]T}$$

After *N* cycles, the accumulated residual viscoelastic strain (ɛ_rve_, *N*) is given by:

(19) $$\varepsilon_{\mathrm{rve},N}=\sum_{i=1}^{N}\varepsilon_{\mathrm{rve},i}=\frac{\pi \sigma_{0}T(1+e^{E_{1}T/\eta_{1}})e^{-E_{1}T/\eta_{1}}}{\eta_{1}(\frac{E_{1}^{2}}{\eta_{1}^{2}}T^{2}+\pi^{2})(1-e^{-E_{1}T/\eta_{1}})}(1-e^{-\frac{E_{1}}{\eta_{1}}NT})$$

The residual strain model without considering damage (ɛp, N)can be obtained as follows:

(20) $$\varepsilon_{p,N}=\varepsilon_{\mathrm{vp},N}+\varepsilon_{\mathrm{rve},N}=\alpha N^{3}+\rho N^{2}+\gamma N+\lambda (1-e^{-\kappa N})$$

$$\begin{array}{l} \mathrm{where}\;\alpha =\frac{\sigma_{0}2aT^{3}}{3\eta_{0}\pi },\;\gamma =\frac{\sigma_{0}(6\pi^{2}T+\pi^{2}aT^{3}-12aT^{3})}{3\eta_{0}\pi^{3}},\;\lambda =\frac{\sigma_{0}\pi T(1+e^{E_{1}T/\eta_{1}})e^{-E_{1}T/\eta_{1}}}{\eta_{1}(\frac{E_{1}^{2}}{\eta_{1}^{2}}+\pi^{2})(1-e^{-E_{1}T/\eta_{1}})},\\ \kappa =\frac{E_{1}}{\eta_{1}}T,\;\rho =\frac{\sigma_{0}bT^{2}}{\eta_{0}\pi } \end{array}$$

During the damage evolution process, the effective area of the material subjected to external loads decreases, which leads to a significant non-linear characteristic in its stress–strain relationship. The above model can more accurately describe the residual strain behavior of asphalt mixtures in an undamaged state. However, it neglects the impact of fatigue damage on the residual strain of asphalt mixtures. Therefore, a residual strain model for asphalt mixtures that considers damage should be established.

During the fatigue damage evolution process, the effective stress applied to the asphalt mixture is:

(21) $$\tilde{\sigma }=\frac{\sigma }{1-D}$$

where $\tilde{\sigma }$ is the effective stress; σ is the Cauchy stress.

By combining the viscoelastic fatigue damage model established in the previous section $D=1-(1-\frac{N}{N_{f}}{)}^{\frac{1}{1+2\beta }}$ with Equations (20) and (21), a residual strain model that accounts for damage can be obtained:

(22) $$\varepsilon_{p,N}=\frac{\varepsilon_{p,N}}{1-D}=\frac{\alpha N^{3}+\rho N^{2}+\gamma N+\lambda (1-e^{-\kappa N})}{{\left(1-\frac{N}{N_{f}}\right)}^{\frac{1}{1+2\beta }}}$$

**Figure 5:**
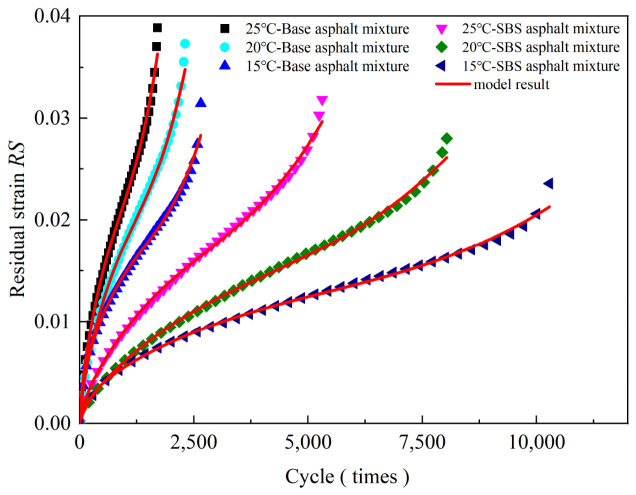
Fitting results of residual strain model of asphalt mixtures.

Compared to the residual strain model that does not account for damage, Equation (22) can describe the effect of fatigue damage on the residual strain of asphalt mixtures. Traditional fatigue damage models can only describe the damage evolution law, while Equation (22) can not only describe the fatigue damage evolution of asphalt mixtures but also accurately capture the evolution of the residual strain. The residual strain curves under different conditions were fitted using Equation (22), with the fitting results shown in Figure 5 and Table 1. From Figure 5 and Table 1, it can be seen that the residual strain model considering damage can more accurately describe the nonlinear evolution of residual strain in SBS-modified and base asphalt mixtures during the damage evolution process.

The traditional modified Burgers model modifies the series Maxwell element in an exponential form as shown in Equation (12), and the resulting residual strain model is referred to as the exponential modified residual strain model, with its simplified form shown in Equation (23). From Equation (23), it can be seen that the exponential modified residual strain model cannot reflect the effect of damage on the residual strain of asphalt mixtures. In this study, a binary function was used to modify the series Maxwell element, and the resulting residual strain model is shown in Equation (22), which is referred to as the binary modified residual strain model.

(23) $$\varepsilon_{p,N}=a(1-e^{-bN})+c(1-e^{-dN})$$

in which *a*, *b*, *c*, *d* are model parameters.

**Figure 6:**
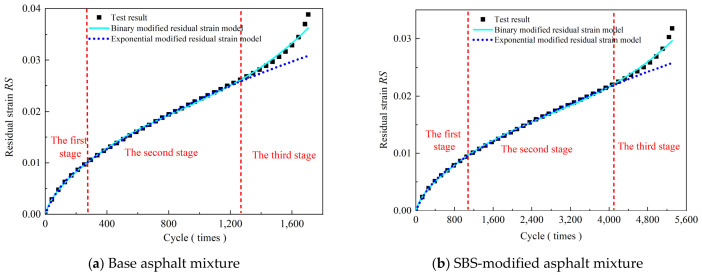
Comparison of calculation results of residual strain models.

Using the base asphalt mixture and SBS-modified asphalt mixture at 25 °C as examples, a comparative analysis of the two models is shown in Figure 6. From Figure 6, it can be seen that both models can accurately describe the residual strain evolution in the first two stages. However, after entering the third stage, the prediction error of the exponential modified residual strain model increases significantly, while the binary modified residual strain model can still accurately describe the residual strain evolution process.

### 2.2. Correlation Analysis Between Fatigue Cracking and Residual Strain Response of SBS-Modified Asphalt Mixture

Under semi-sinusoidal cyclic loading, both SBS-modified and base asphalt mixtures accumulate residual strain, during which fatigue cracking continuously occurs. Although the accumulation of residual viscoelastic strain, viscoplastic strain, and fatigue cracking are different physical processes, all three can occur simultaneously under the action of the load. Therefore, it is necessary to analyze the correlation between fatigue cracking and residual strain response in SBS-modified asphalt mixtures.

**Figure 7:**
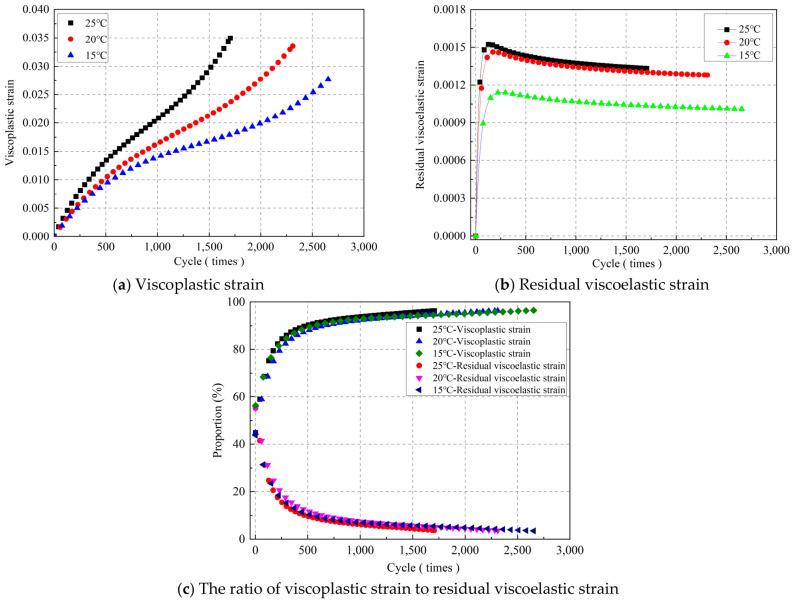
Viscoplastic strain and residual viscoelastic strain of base asphalt mixtures.

**Figure 8:**
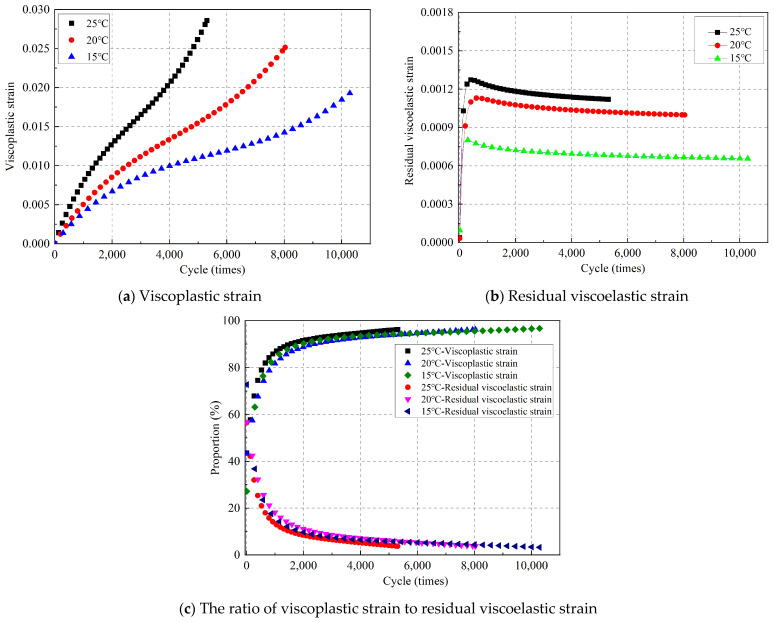
Viscoplastic strain and residual viscoelastic strain of SBS-modified asphalt mixtures.

Residual strain is mainly composed of residual viscoelastic strain and viscoplastic strain. During the fatigue cracking process, the variation patterns of residual viscoelastic strain and viscoplastic strain in SBS-modified and base asphalt mixtures are shown in Figure 7 and Figure 8. As can be seen from Figure 7 and Figure 8, both residual viscoelastic strain and viscoplastic strain are generally positively correlated with temperature.

As shown in Figure 7a,b and Figure 8a,b, during the propagation of fatigue cracks, the residual viscoelastic strain rapidly increases to a peak level only during a short initial stage, with the rate of increase gradually decreasing. Subsequently, the residual viscoelastic strain decreases at a relatively slow rate, but the magnitude of this decrease is very limited. This is mainly because the damage-free stage experienced by the asphalt mixture is very brief, after which part of the viscoelastic strain recovers, leading to a gradual decline in residual viscoelastic strain. Fatigue cracking continues to occur even when residual viscoelastic strain no longer accumulates and begins to decline, indicating that the correlation between fatigue cracking and the accumulation of residual viscoelastic strain is very weak. During the propagation of fatigue cracks, viscoplastic strain continuously undergoes nonlinear accumulation, exhibiting a nonlinear evolution trend that is more closely aligned with that of the total residual strain.

When residual viscoelastic strain no longer accumulates, fatigue cracking and viscoplastic strain accumulation in both SBS-modified and base asphalt mixtures continue to occur simultaneously, indicating a strong correlation between fatigue cracking and viscoplastic strain in asphalt mixtures. At the same time, as shown in Figure 7c and Figure 8c, which illustrate the proportion of residual viscoelastic strain and viscoplastic strain, the proportion of residual viscoelastic strain is relatively large in the initial stage of damage evolution, but this proportion gradually decreases as fatigue cracking progresses, falling below 6% near the end of loading. In contrast, the proportion of viscoplastic strain gradually increases, exceeding 94% at the end of loading. From the above analysis, it can be concluded that fatigue cracking in both SBS-modified and base asphalt mixtures is strongly correlated with viscoplastic strain, whereas its correlation with residual viscoelastic strain is very weak.

**Figure 9:**
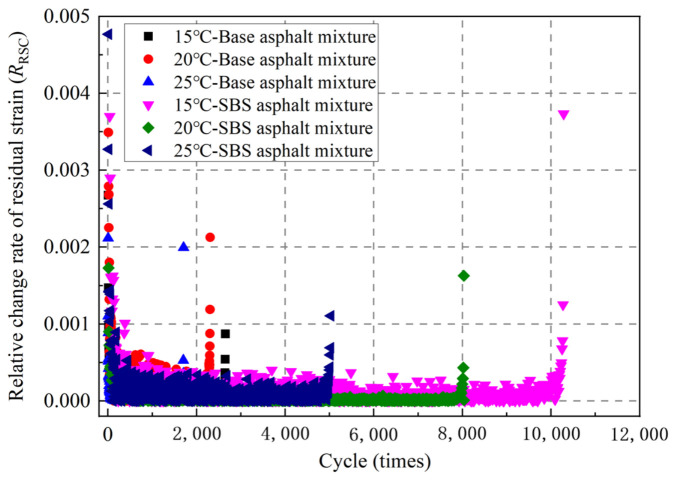
Ratio of residual strain change of asphalt mixtures.

To more accurately analyze the residual strain response of asphalt mixtures during fatigue cracking, the relative rate of residual strain change was defined as shown in Equation (25). The curve of the relative rate of residual strain change obtained from Equation (25) is presented in Figure 9. As shown in Figure 9, the relative rate of residual strain in asphalt mixtures exhibits a nonlinear three-stage trend, namely: a rapid decrease stage, a stable stage, and a rapid increase stage. It should be noted that during cyclic loading, both fatigue damage softening and strain hardening mechanisms occur simultaneously. When the strain hardening mechanism dominates, the rate of residual strain decreases; when the two mechanisms are comparable, the rate of residual strain remains relatively stable; and when fatigue damage softening dominates, the rate of residual strain increases. The three-stage trend of the relative rate of residual strain is the result of the combined effect of these two mechanisms. The strain hardening mechanism dominates the initial stage, leading to the rapid decrease stage. Subsequently, the fatigue damage softening mechanism gradually strengthens and eventually becomes dominant, resulting in the stable stage and the rapid increase stage, respectively.

Since residual strain and fatigue cracking occur simultaneously and are correlated, the relative rate of residual strain change can, to some extent, reflect the asphalt mixture’s ability to resist residual strain and fatigue cracking. As shown in Figure 9, initially, the relative rate of residual strain changes rapidly from a high level to a low level, indicating that the asphalt mixture has weak resistance to residual strain in the early stage of damage evolution. However, the asphalt mixture still maintains strong resistance to fatigue cracking during this phase. Then, the relative rate of residual strain enters a stable phase, maintaining a low, stable level over the long term, which suggests that the mixture’s ability to resist residual strain and fatigue cracking is relatively stable during this stage, and fatigue damage evolves steadily. Finally, the relative rate of residual strain enters a rapid increase phase. The residual strain of both SBS-modified and base asphalt mixtures accumulates quickly, and the overall fatigue cracking resistance of the specimens rapidly decreases because fatigue damage gradually evolves to the failure threshold. From the above analysis, it can be concluded that the relative rate of residual strain change can reasonably reflect the overall fatigue cracking and residual strain resistance of the asphalt mixture. The faster the relative rate of residual strain increases, the faster the asphalt mixture experiences fatigue failure, meaning the shorter the fatigue life.

Further analysis of Figure 9 shows that under the same stress ratio and loading temperature conditions, the relative rate of residual strain change in SBS-modified asphalt mixtures enters the rapid increase phase later than that in the base asphalt mixtures. This indicates that the SBS modifier can form a three-dimensional physical gel structure inside the overall material structure of the asphalt mixture and enhance its ability to resist fatigue cracking, which is consistent with the reported ability of SBS to form a three-dimensional physical gel structure.

### 2.3. Nonlinear Fatigue Damage Evolution Analysis of SBS-Modified Asphalt Mixture

**Figure 10:**
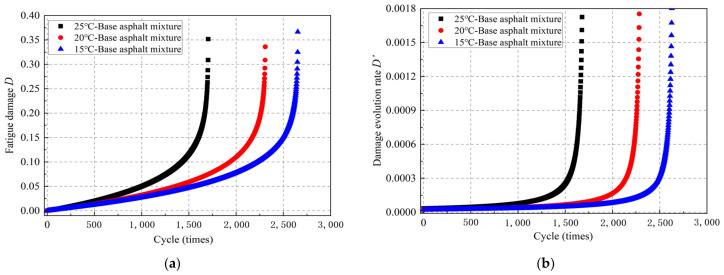
Fatigue damage of base asphalt mixtures under different temperatures. (**a**) Fatigue damage curve of the base asphalt mixture. (**b**) Fatigue damage evolution rate curve of the base asphalt mixture.

**Figure 11:**
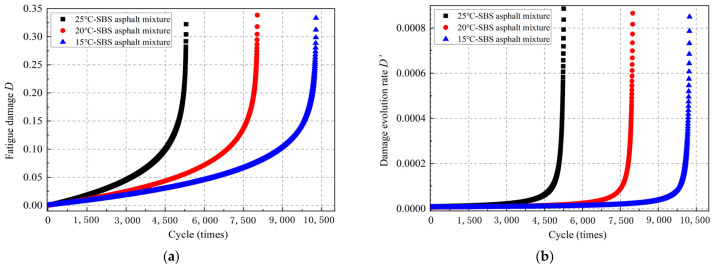
Fatigue damage of SBS-modified asphalt mixtures under different temperatures. (**a**) Fatigue damage curve of SBS-modified asphalt mixture. (**b**) Fatigue damage evolution rate curve of SBS-modified asphalt mixture.

By substituting the obtained damage model parameters into the fatigue damage model, the fatigue damage evolution of the asphalt mixture can be described. The fatigue damage curves under different conditions are shown in Figure 10 and Figure 11.

As seen from Figure 10 and Figure 11, the fatigue damage evolution of the asphalt mixture follows a nonlinear evolution trend with a gradually increasing rate [22,24]. When the test temperature increases from 15 °C to 25 °C, the accumulated fatigue damage for the same loading cycles gradually increases, meaning that the damage evolution rate is positively correlated with temperature. This is because, at higher test temperatures, the asphalt mixture exhibits a larger phase angle, and the material’s viscoelastic mechanical properties become more pronounced. In this case, fatigue cracks can propagate at a faster rate, leading to more ductile fractures. The crack distribution is less concentrated, and the crack size is relatively larger [19].

As shown in Figure 10 and Figure 11 when the testing temperature is relatively low, the elastic mechanical properties of the asphalt mixture are more pronounced, the fatigue cracking rate is slower, the accumulated fatigue damage per load cycle is reduced, and the fatigue damage evolution rate is smaller. Under these conditions, brittle fracture is more likely to occur, where the cracks are concentrated and fine in size. Moreover, compared to the base asphalt mixture, the SBS-modified asphalt mixture exhibits a smaller fatigue damage evolution rate. This is because, compared to the base asphalt mixture, the SBS modifier forms a three-dimensional physical gel structure in the asphalt mixture, effectively enhancing the mixture’s ability to resist fatigue cracking. This reduces the accumulated fatigue damage per load cycle, causing the material’s phase angle to decrease and its elastic mechanical response characteristics to become more pronounced, thereby lowering the fatigue damage evolution rate.

## 3. Conclusions

By establishing a residual strain model for SBS-modified asphalt mixtures considering damage, the correlation between fatigue cracking and residual strain response of SBS-modified asphalt mixtures was analyzed, and the nonlinear fatigue damage evolution law was described. The main conclusions are as follows:

(1) The accumulation of residual strain and fatigue cracking in SBS-modified asphalt mixtures under half-sine cyclic loading occur simultaneously. The established residual strain model can accurately describe the corresponding residual strain response and nonlinear fatigue damage evolution law.

(2) The residual strain of SBS-modified asphalt mixtures mainly consists of viscoplastic strain and residual viscoelastic strain. There is a strong correlation between fatigue cracking and viscoplastic strain, but the correlation with residual viscoelastic strain is very weak.

(3) The defined relative change rate of residual strain can reasonably reflect the ability of SBS-modified asphalt mixtures to resist fatigue cracking and residual strain. The faster the relative change rate of residual strain increases, the more rapidly fatigue failure occurs in the asphalt mixture.

(4) At relatively high temperatures, SBS-modified asphalt mixtures exhibit pronounced viscous mechanical characteristics, rapid fatigue damage evolution, and fatigue cracks that are widely distributed and coarse in size. In contrast, at lower temperatures, their elastic mechanical characteristics are more pronounced, fatigue damage evolves more slowly, and fatigue cracks are concentrated and finer in size.

(5) The macroscopic test results in this study show that the SBS modifier significantly improves the resistance of asphalt mixtures to residual strain accumulation and fatigue cracking. Combined with existing theoretical analyses, it is inferred that the three-dimensional physical gel network formed by SBS polymer is the main potential mechanism responsible for the performance improvement.

In future research, microstructural characterization methods such as fluorescence microscopy, AFM, and DSR rheological tests can be further adopted to quantitatively reveal the relationship between the 3D physical gel network of SBS and the macroscopic nonlinear fatigue damage evolution, so as to establish a more comprehensive multi-scale mechanism for SBS-modified asphalt mixtures. The high fitting degree of the model to the modeling data alone is not enough to prove its predictive ability. This study has not yet been verified by independent data sets under different stress levels, loading frequencies or temperature conditions, which constitutes the main limitation of this work. In the follow-up study, we plan to introduce fatigue test data with different stress ratios and loading frequencies to independently verify the model and further improve its generalization ability. The established residual strain model considering damage contains many parameters, such as *α*, *ρ*, *γ*, *λ*, *κ*, *β*. In this paper, only β is used to discuss the damage evolution characteristics of the asphalt mixture. In the future, the relationship between other parameters and valuable information on the inner structure and mechanical properties of the samples will be further studied.

## 4. Materials and Methods

### 4.1. Asphalt Binder

Styrene-butadiene-styrene (SBS) modified asphalt is widely utilized in road engineering due to its enhanced properties. In this study, SBS-modified asphalt is selected as the primary material, with unmodified matrix asphalt serving as the control. Commercial SBS-modified asphalt (SBS-modified asphalt binder is about 4.5%) has been widely used in paving asphalt pavement all over the world. This commercial SBS-modified asphalt is used in this study. SBS-modified asphalt is a composite material formed through the physical blending of base asphalt and the SBS modifier. The SBS modifier creates a complex polymeric network within the base asphalt, forming a three-dimensional physical gel structure that significantly improves the material’s mechanical properties. The asphalt used in this study is the SBS-modified asphalt commonly employed in the Hong Kong region, with its technical specifications outlined in Table 2. The SBS modifier is a block copolymer, with polystyrene segments at the ends physically crosslinking to form an aggregated phase in the asphalt, while the polybutadiene mid-segment forms a continuous phase. This results in a thermoreversible three-dimensional physical gel network. The physical gel structure imparts remarkable elastic recovery and deformation resistance to the SBS-modified asphalt, enabling it to exhibit superior elastic mechanical responses under cyclic loading compared to unmodified base asphalt. This significantly delays the accumulation of residual strain and the progression of fatigue damage.

### 4.2. Specimen Preparation

**Figure 12:**
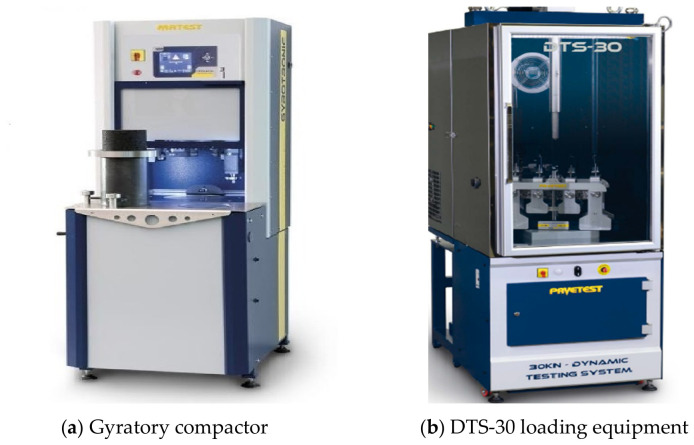
Gyratory compactor and DTS-30 loading equipment.

Specimens were fabricated using a rotary compactor (Figure 12a), selected for its ability to produce asphalt mixtures with superior homogeneity and reproducibility. Indirect tensile fatigue tests were conducted on these specimens using a Dynamic Test System-30 (DTS-30, Figure 12b). Cyclic loading was applied at a frequency of 10 Hz, corresponding to vehicular speeds of 60–65 km/h. Testing was performed under intermediate temperatures prone to fatigue cracking—15 °C, 20 °C, and 25 °C. For each condition, five replicate tests were conducted to ensure statistical reliability.

**Figure 13:**
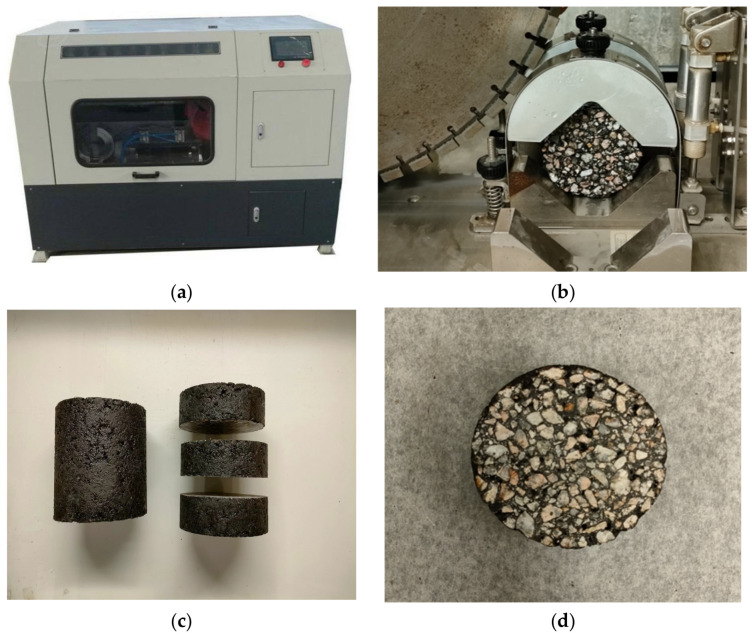
Asphalt mixture specimens cutting. (**a**) Specimen cutting machine. (**b**) Specimen cutting process. (**c**) Comparison before and after specimen cutting. (**d**) Test specimen.

Granite, a material commonly utilized in Hong Kong, served as the aggregate, configured according to the gradation of Stone Mastic Asphalt (SMA), a formulation widely adopted in road engineering applications (Table 3). The asphalt binder content was set at 6%, with target air voids of 4% (±0.5%). Cylindrical specimens measuring 150 mm in height and 100 mm in diameter were compacted using a rotary compactor (Figure 12a). Subsequently, as illustrated in Figure 13, the top and bottom 15 mm of each specimen were first removed using a dedicated cutting apparatus, resulting in an intermediate specimen of 120 mm in height. Then, the top and bottom 40 mm of this intermediate specimen were further cut off, finally yielding test specimens with dimensions of 100 mm in diameter and 40 mm in height.

### 4.3. Indirect Tensile Strength Testing

**Figure 14:**
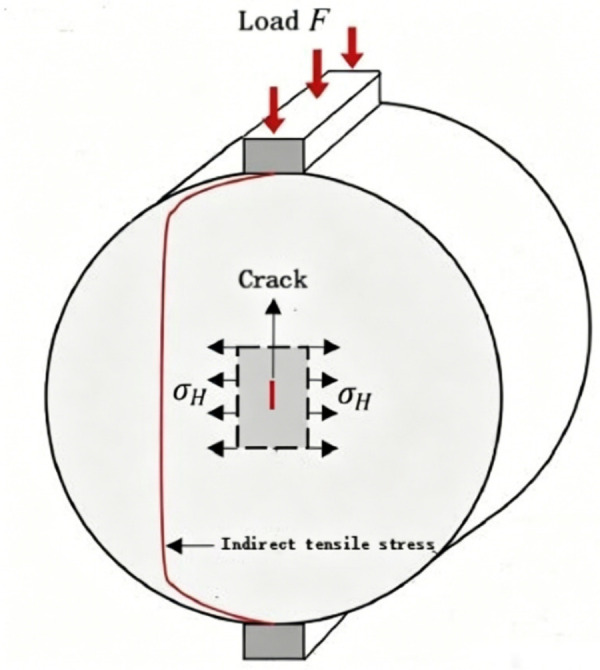
Schematic of indirect tensile test.

The indirect tensile fatigue (ITF) test, illustrated in Figure 14, enables precise measurement of asphalt mixture strain responses under cyclic loading using displacement sensors. ITF testing offers notable advantages, including simple specimen preparation, strong repeatability, and ease of implementation in engineering practice, and has therefore been widely adopted in the field of pavement engineering. Accordingly, this study employs ITF testing to investigate the fatigue damage characteristics of asphalt mixtures.

**Figure 15:**
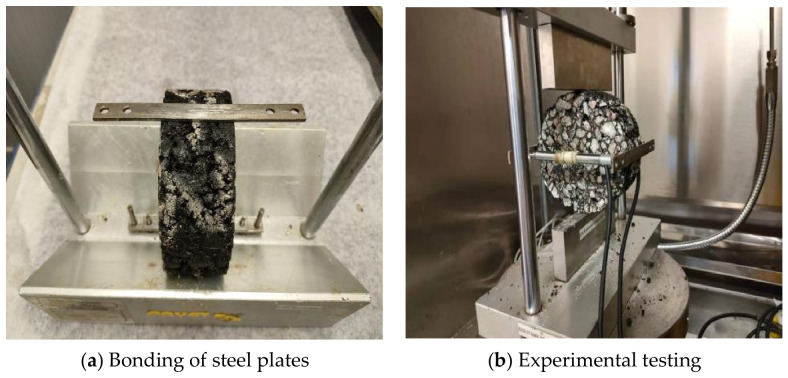
Indirect tensile tests of asphalt mixtures.

Prior to conducting indirect tensile fatigue (ITF) tests, specimens were subjected to indirect tensile strength (ITS) testing to determine the maximum load at failure, which serves as the reference for setting the fatigue load amplitude. ITS tests were performed under displacement-controlled, constant-rate loading at 50 mm/min. The primary procedure followed established protocols:

(1) Two perforated steel plates were symmetrically bonded to the asphalt mixture specimen along its height using an epoxy adhesive, as shown in Figure 15a.

(2) Displacement sensors were installed through the steel plate apertures to capture the transverse tensile strain response of the specimen during loading with the DTS-30 device (Figure 15b).

(3) The specimen was carefully centered on the supports and conditioned in a temperature-controlled chamber for 2 h.

(4) Axial load was applied at a constant displacement rate of 50 mm/min using the DTS-30, until specimen failure, with the peak load (*F*_max_) recorded.

The indirect tensile stress of the specimen was approximated using the following equation, with the stress corresponding to *F*_max_ defined as the indirect tensile strength.

(24) $$\sigma =\frac{2F}{\pi \;t\;\Omega }$$

In the equation, *F* is the applied force (N), *t* is the specimen height (mm), and *Ω* is the specimen diameter (mm).

### 4.4. Indirect Tensile Fatigue Testing

**Figure 16:**
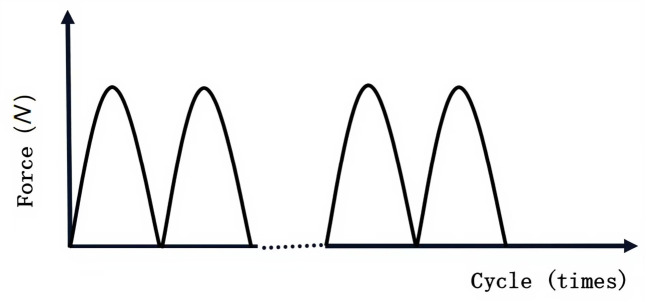
Loading waveform schematic of indirect tensile fatigue tests asphalt mixtures.

Indirect tensile fatigue (ITF) tests were conducted on asphalt mixture specimens to investigate their fatigue damage characteristics under cyclic loading. A schematic of the constant stress amplitude loading waveform is shown in Figure 16. The load was applied as a half-sine cyclic waveform under stress-controlled conditions.

In fatigue testing of asphalt mixtures, selecting an appropriate stress level is crucial for the smooth progression of the experiment, as the stress level directly influences the fatigue life of the asphalt mixture. When the stress ratio is too high (e.g., greater than 0.5), it leads to an overly short fatigue life, which significantly deviates from the actual fatigue life of road surfaces, and results in a higher degree of dispersion in the test outcomes. Conversely, when the stress ratio is too low (e.g., less than 0.1), it results in excessively long loading times, and may even prevent fatigue failure of the specimen, while the loading equipment is prone to stalling, leading to lower repeatability of the test. Considering both the stability of the test data and the time factor, and based on experimental results from relevant studies, this study sets the stress ratio at 0.3, with the load amplitude being 0.3*F*_max_.

The DTS-30 was used to apply cyclic loading to the asphalt mixture specimens until fatigue failure occurred. The DTS-30 data acquisition system can record the strain results of the asphalt mixture at the end of each loading cycle, i.e., the residual strain (RS). Under half-sine cyclic loading, the asphalt mixture undergoes cumulative creep deformation accompanied by the evolution of fatigue damage. During this process, fatigue cracking and strain accumulation occur simultaneously. Although it is difficult to precisely separate the two, phenomenological methods, such as digital image correlation, can effectively relate them. The studies by Jiang [15,16] indicate that under half-sine cyclic loading, fatigue damage and residual strain of asphalt mixtures exhibit a strong correlation, and analyzing the residual strain response allows the fatigue damage evolution to be captured with reasonable accuracy.

**Figure 17:**
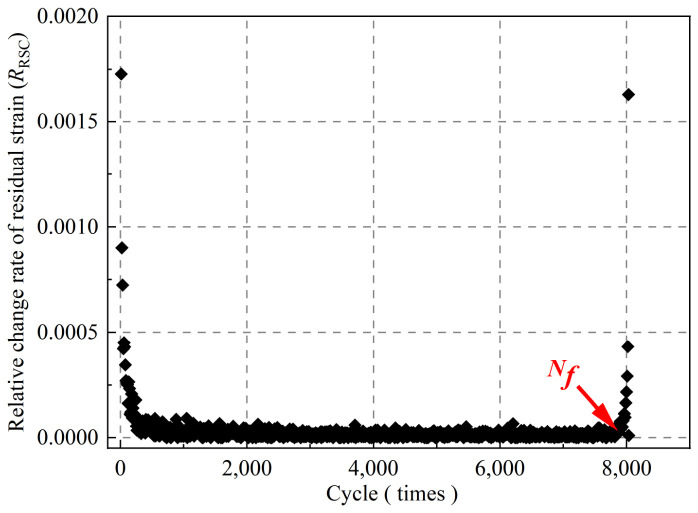
Typical curve of ratio of residual strain change of asphalt mixture.

To more accurately analyze the residual strain response of the asphalt mixture during the damage evolution process, the Ratio of Residual Strain Change (*R*_RSC_) is defined as shown in Equation (25). The *R*_RSC_ curve for the asphalt mixture obtained from Equation (25) is presented in Figure 17. As shown in Figure 17, the *R*_RSC_ of the asphalt mixture under half-sine cyclic loading exhibits a nonlinear three-stage variation trend. The intersection of the *R*_RSC_ curves in the second and third stages represents the point at which the asphalt specimen, due to accumulated fatigue damage, has almost lost its ability to resist fatigue cracking. The load cycle at this intersection is defined as the fatigue life. The fatigue life results of the asphalt mixture under different working conditions are shown in Table 4.

(25) $$R_{RSC}=\frac{PS_{N+1}-PS_{N}}{PS_{N}}$$

In the equation, *PS_N_* represents the residual strain of the N-th cycle.

## Figures and Tables

**Table 1:** Fitting results of residual strain model coupling damage.

Test Number	*α*	*ρ*	*γ*	*λ*	*κ*	*β*	*R* ^2^
1	2.19 × 10^−5^	−1.14 × 10^−8^	2.65 × 10^−12^	0.00101	0.01707	8.13260	0.998
2	2.39 × 10^−5^	−1.16 × 10^−8^	3.25 × 10^−12^	0.00128	0.02336	8.11030	0.997
3	3.38 × 10^−5^	−2.21 × 10^−8^	8.41 × 10^−12^	0.00133	0.03161	8.08222	0.997
4	4.32 × 10^−6^	−5.83 × 10^−10^	3.50 × 10^−14^	0.00071	0.00441	8.90380	0.995
5	5.18 × 10^−6^	−7.23 × 10^−10^	5.83 × 10^−14^	0.00100	0.00686	8.70471	0.987
6	8.90 × 10^−6^	−1.88 × 10^−9^	2.29 × 10^−13^	0.00112	0.01040	8.45255	0.971

**Table 2:** Technical properties of asphalt.

Parameter	SBS-Modified Asphalt	Base Asphalt
Penetration (25 °C) (0.1 mm)	57	69
Kinematic Viscosity (135 °C) (Pa·s)	1.84	1.54
Flash Point (°C)	308	315
Solubility (%)	96.83	99.97
Softening Point (°C)	54.9	44.2
Ductility (5 °C, 5 cm/min) (cm)	33.4	27
Elastic Recovery (25 °C) (%)	93	87

**Table 3:** Aggregate gradation.

Component	Relative Density (g/cm^3^)	Size (mm)	Percentage (%)
Coarse Aggregate	2.642	14–10	3.5
	2.663	10–5	59.5
	2.709	5–2.36	9.0
Fine Aggregate	2.649	2.36–0.075	16.5
Filler	2.661	<0.075	9.5
Lime	2.587		2.0

**Table 4:** Fatigue life of indirect tensile fatigue tests with constant stress amplitude of asphalt mixtures.

Asphalt Mixture	Test No.	Sample 1	Sample 2	Sample 3	Sample 4	Sample 5	Mean Value	Standard Deviation	Coefficient of Variation(%)	Confidence Interval
base asphalt Mixture	1	2423	2915	2589	2731	2607	2653	283.1	10.672	[2301, 3005]
	2	2102	2493	2216	2395	2344	2310	207	8.971	[2053, 2567]
	3	1522	1873	1634	1781	1715	1705	168.6	9.891	[1495, 1915]
SBS-Modified Asphalt Mixture	4	9315	11,243	9976	10,592	10,209	10,285	903.1	8.781	[9163, 11,407]
	5	7216	8823	7805	8362	7979	8035	858.1	10.679	[6968, 9102]
	6	4812	5793	5126	5459	5340	5306	492.3	9.279	[4694, 5918]

## Data Availability

Data are contained within the article. Some or all of the data, models, or code that support the findings of this study are available from the corresponding author upon reasonable request.
